# Arbuscular Mycorrhizal Fungi (*Glomus mosseae*) Improves Growth, Photosynthesis and Protects Photosystem II in Leaves of *Lolium perenne* L. in Cadmium Contaminated Soil

**DOI:** 10.3389/fpls.2018.01156

**Published:** 2018-08-13

**Authors:** Huihui Zhang, Nan Xu, Xin Li, Jinghong Long, Xin Sui, Yining Wu, Jinbo Li, Jifeng Wang, Haixiu Zhong, Guang Y. Sun

**Affiliations:** ^1^School of Resources and Environmental Science, Northeast Agricultural University, Harbin, China; ^2^Institute of Natural Resources and Ecology, Heilongjiang Academy of Sciences, Harbin, China; ^3^College of Life Sciences, Heilongjiang University, Harbin, China; ^4^College of Life Science, Northeast Forestry University, Harbin, China

**Keywords:** *Lolium perenne* L., cadmium (Cd), arbuscular mycorrhizal fungi, photosynthetic characteristics, PSII

## Abstract

In this study, the effects of inoculating arbuscular mycorrhizal fungi (*Glomus mosseae*) on the growth, chlorophyll content, photosynthetic gas exchange parameters, and chlorophyll fluorescence characteristics of *Lolium perenne* L. in cadmium (Cd) contaminated soil were investigated. The results showed that the root vigor of *L. perenne* declined, while the chlorophyll content significantly decreased with the increase of Cd content, especially the chlorophyll a content in leaves. The photosynthetic carbon assimilation capacity and PSII activity of *L. perenne* leaves were also significantly inhibited by Cd stress, especially the electron transfer at the receptor side of PSII, which was more sensitive to Cd stress. The infection level of *G. mosseae* on *L. perenne* roots was relatively high and inoculation with *G. mosseae* increased the mycorrhizal infection rate of *L. perenne* roots up to 50–70%. Due to the impact of the mycorrhizal infection, the Cd content in *L. perenne* roots was significantly increased compared to non-inoculated treatment; however, the Cd content in the aboveground part of *L. perenne* was not significantly different compared to the non-inoculated treatment. After inoculation with *G. mosseae*, the root vigor of *L. perenne* increased to some extent, alleviating the chlorophyll degradation in *L. perenne* leaves under Cd contaminated soil. Infection with *G. mosseae* can improve the stoma limitation of *L. perenne* leaves in Cd contaminated soil and increase the non-stomatal factors including the tolerance of its photosynthetic apparatus to Cd, to improve photosynthetic capacity. *G. mosseae* infection can improve the photosynthetic electron transport capacity of PSII in *L. perenne* leaves under Cd stress and promotes the activity of the oxygen-evolving complex to different degrees at the donor side of PSII and the electron transport capacity from Q_A_ to Q_B_ on the receptor side of PSII. Thus, this guarantees that *L. perenne* leaves inoculated with *G. mosseae* in Cd contaminated soil have relatively higher PSII activity. Therefore, inoculation with *G. mosseae* can improve the capacity of Cd tolerance of *L. perenne* with regard to various aspects, such as morphological characteristics and photosynthetic functions, and reduce the toxicity of Cd on *L. perenne*.

## Introduction

Over the past 50 years, global cadmium (Cd) emission in the environment reached 2.2 × 10^4^ t (Singh et al., 2003). Cd is an unnecessary and toxic element for living organisms, and when humans intake excess Cd, functional impairment of the kidneys and osteoporosis will be induced (Alfvén et al., 2000). In addition, Cd has carcinogenic, teratogenic, and mutagenic effects on human beings (Qian et al., 2009). Cd influences the physiological and biochemical processes of plants, such as inhibiting seed germination and plant growth (Peralta et al., 2001; Rizwan et al., 2017), affecting the uptake of mineral elements (Murtaza et al., 2017; Qaswar et al., 2017), resulting in leaf necrosis (Ciecko et al., 2001; López-Millán et al., 2009), restraining nutrient absorption (Hall, 2002; Dong et al., 2006), changing the structure and function of mitochondria, blocking the synthesis of carbohydrates and proteins (Kieffer et al., 2008), causing a disorder of the hormonal metabolism (Masood et al., 2012), increasing reactive oxygen species (Zhang et al., 2007), and causing leakage of cellular electrolytes (Rodrıguez-Serrano et al., 2006; Gill et al., 2015). Cd stress influences plant photosynthetic capacity (Kola and Wilkinson, 2005). Studies have indicated that Cd stress has resulted in the destruction of the chlorophyll structure in *Populus deltoides* × *P. nigra* leaves, and the number of chloroplasts declined, the chloroplast membrane swelled, the membrane structure blurred and even disappeared, and the thylakoids became disordered (Zhang et al., 2014). Cd also leads to the blockage of plant solar energy utilization (Krantev et al., 2008) and affects the carbon assimilation process (Xue and Gao, 2017). However, previous studies have found that low concentrations of Cd stress result in the increase of the large and small subunits of Rubisco, as well as increase Rubisco activity in *Typha angustifolia* leaves (Bah et al., 2010) and the activities of relevant photosynthetic carbon assimilation enzymes of *Arabidopsis thaliana* (Semane et al., 2010) and *Glycine max* L. (Hossain et al., 2012). However, relatively high concentrations of Cd could combine with the functional groups of some enzymes to inhibit their activities (Tukaj et al., 2007). For example, the content and activity of Rubisco in *Populus tremula* L. leaves obviously declined under Cd stress (Marmiroli et al., 2013).

Arbuscular mycorrhizal fungi (AMF), which obtain nutrients through infecting host plant root systems and are the most widely distributed symbiont, can infect the root systems of over 80% of vascular plants (Li, 1998). The hypha of AMF can serve as an important channel for soil nutrients entering plants, hence it can also promote the absorption of nutrients by host plants (Karagiannidis et al., 2002), promote plant growth, yield, and quality (Bowles et al., 2016; Rozpądek et al., 2016), improve plant photosynthetic capacity and PSII function (Chen et al., 2017a; Mathur et al., 2018), as well as accelerate plant growth and enhance plant stress resistance through impacting root exudation (Chen et al., 2017b). Some studies also found that AMF could improve plant tolerance to heavy metal pollution (Del et al., 1999a,b). Under heavy metal stress, through chelation and hyphae immobilization (Gonzalez-Chavez et al., 2002; Rufyikiri et al., 2004) or the complexes formed by cysteine in the mycorrhiza combined with heavy metals, AMF can retain heavy metals within roots (Dehn and Schüepp, 1990), inhibiting heavy metal transport from roots to the aboveground parts of the plant, effectively reducing heavy metal toxicity and promoting plant growth (Tullio et al., 2003; Christie et al., 2004). Pallara et al. (2013) selected *Populus alba* as the experiment material and found that inoculation with *Glomus mosseae* was beneficial for inducing the expression of genes encoding chelate synthetase in leaves and controlling cellular antioxidant levels, thus improving host plant tolerance to heavy metals. In addition, AMF hyphae had a relatively higher cation exchange capacity and heavy metal adsorption capacity, which helped plants infected by AMF to achieve improved heavy metal tolerance (Joner et al., 2000).

*Lolium perenne* L. is an annual graminaceous monocot plant that is easily grown and has a relatively large biomass, strong regeneration capacity, and pest resistance; it is an important cultivated forage plant and green manure plant with a high economic and ecological value (Hebeisen et al., 2010; Chang et al., 2017). Moreover, *L. perenne* has been shown to have very strong resistance to heavy metals and can concentrate heavy metals (Lial and Huang, 2002). Therefore, *L. perenne* is commonly used as pasture and grass for golf courses and is also used for the phytoremediation of areas with heavy metal contamination. Many relevant studies have shown that *L. perenne* could promote plant resistance to heavy metals. For example, the inoculation of AMF can improve the plant capacity for nutrient adsorption including in *Astragalus sinicus* L. and *Zea mays* L (Chen and Zhao, 2009; Zhang et al., 2010). It can also increase the retention of heavy metals in plant roots and decrease the distribution of heavy metals in the aboveground part of host plants (Lins et al., 2006; Słomka et al., 2011), or decrease the heavy metal content in host plants through improving host plant growth, while increasing biomass (Andrade et al., 2009). Previous studies have found that inoculation with AMF could improve plant absorption of heavy metals (Aloui et al., 2012). However, few studies have been conducted on the effects of AMF on the photosynthetic characteristics of *L. perenne* in Cd contaminated soil. Thus, this research intended to study the effects of AMF (*G. mosseae*) inoculation on the growth, chlorophyll content, photosynthetic gas exchange parameters, and chlorophyll fluorescence parameters of *L. perenne* in soil contaminated by different Cd concentrations from the perspective of plant photosynthetic mechanisms, with the purpose of providing fundamental data for revealing the mechanism of AMF improving *L. perenne* resistance to Cd stress.

## Materials and Methods

### Experimental Materials

The experiments were conducted in the Laboratory of Soil Science, Northeast Agricultural University (Harbin, Heilongjiang Province, China) between March and June, 2016. The culture medium utilized well-mixed turfy soil and cleaned river sand at a ratio of 1:1 (v/v), which was sterilized in a high-pressure sterilizer at 121°C for 2 h to kill native mycorrhiza and other microbes in the soil. The mycorrhizal fungi used in this study were *G. mosseae*, and the inoculum was purchased from the Chinese Arbuscular Mycorrhizal Fungi Germplasm Resources Information System, labeled as “BGCAH01.” After propagation for four months using *Trifolium repens* L. as host plant, the soil containing fungal spores, hyphae, and the infected root section of the host plant was used as inoculum. The plant species used was *L. perenne*, which was provided by the Heilongjiang Academy of Agricultural Sciences. Before use, the seeds were immersed in an H_2_O_2_ solution with volume percent fraction of 10% for 10 min to kill infectious microbes on the surface of the seeds. The seeds were then washed with sterilized water and processed to accelerate germination in a sterile culture dish in a manual climatic box with conditions as follows: temperature of 25/23°C (light/dark), light intensity of 400 μmol⋅m^-2^⋅s^-1^, photoperiod of 12/12 h (light/dark), and relative humidity of approximately 75%.

Different Cd levels were tested including 0, 30, 90, and 180 mg⋅kg^-1^. Under each Cd concentration, two treatments were conducted: one treatment inoculated with *G. mosseae* (+AMF) and the other without AMF inoculation (CK). In the +AMF treatment, 50 g of soil containing *G. mosseae* were added into every 1 kg of culture matrix; while in the CK treatment, the same amount of sterile soil was added into the matrix. After the sterilized soils with/without fungi were mixed well with the respective treated matrixes, they were used to fill in the cultivation pots (completely intermixed). The seeds processed with accelerated germination were planted evenly in culture pots with a diameter of 12 cm and a height of 15 cm. About 50 seeds were sown in each pot, and 0.5 cm of sterile soil covered the soil surface. Each treatment consisted of 5 pot replicates, with a total of 40 pots. The culture pots were placed in a manual climatic box set to 25°C, with a light intensity of 400 μmol⋅m^-2^⋅s^-1^, photoperiod of 12/12h (light/dark), and relative humidity of 75%. The plants were irrigated and seedling management was conducted regularly. The relative water content was maintained at approximately 80% in the soil in the pots containing different treatments. When the seedling height reached about 20–30 cm, and after significant differences in plant phenotype appeared between various treatments, relevant data was measured.

### Parameter Measurements and Methods

#### Measurement of Growth Parameters

After the plant height was measured, the plants were removed from the culture matrix. The root length of each plant was measured after the culture matrix on the root surface was removed. Any remaining water on the root surface was wiped with absorbent paper, the aboveground and underground parts were placed into an aluminum box respectively, at 105°C for 30 min, and then dried at 60°C for 30 h until a constant weight was reached; then, the biomass was weighed. The aboveground biomass, underground biomass, and root to shoot ratio of each plant were calculated, wherein the root to shoot ratio = underground biomass/aboveground biomass.

#### Measurement of Mycorrhizal Infection Rate, Chlorophyll Content, and Root Vigor

The Trypan blue staining method was used to measure the mycorrhizal infection and after dyeing, the ratio of infected roots (i.e., mycorrhizal infection rate) was measured under a microscope using the modified cross-bonded method. Mycorrhizal infection rate (%) = the root with arbuscules/measured root amount × 100. The acetone-ethanol solvent extraction was applied for the determination of chlorophyll content. The fully expanded top leaves of *L. perenne* in each treatment were selected. The collected *L. perenne* leaves were soaked in a 1:1 mixture of acetone and ethanol in darkness and the samples were shaken periodically until the green completely faded from the leaves. The absorbance values at 663, 646, and 470 nm were determined, and the calculation of chlorophyll content was conducted according to the method described by Porra (2002). Root vigor was measured following the TTC method (Shao et al., 2009). The dry samples in the aboveground and underground parts of *L. perenne* were digested using a Microwave Digestion System (MARS6, American CEM) with nitric acid digestive solution until the samples were a clear liquor. Then, samples were diluted to a constant volume, and an atomic absorption spectrophotometer (AA7700, SHIMADZU, Japan) was applied to determine the Cd content.

#### Measurement of Photosynthetic Gas Exchange Parameters

The measurement of photosynthetic parameters was carried out between 9:00 a.m. and 11:00 a.m. The CIRAS-2 portable photosynthesis system (PPsystem company, United Kingdom) was used to measure photosynthetic parameters such as net photosynthetic rate (*P*_n_), stomatal conductivity (*G*_s_), transpiration rate (*T*_r_), and intercellular CO_2_ concentration (*C*_i_) of fully expanded leaves located at the top of each treated plant. The leaves were tiled to fill the whole leaf chamber during determination. During the measurements, the CIRAS-2 self-made light source and built-in CO_2_ cylinder were used, the light intensity was set to 800 μmol⋅m^-2^⋅s^-1^, and the CO_2_ concentration within the fixation system was 400 μL⋅L^-1^.

#### Measurement of Chlorophyll Fluorescence Parameters

Fully expanded leaves at the top of each treated plant were selected and the leaves were processed with 30-min dark adaptation using the dark adaptation clips. Then, the Handy-PEA multi-function plant efficiency analyzer (Hansatech Company, United States) was used to measure the OJIP curves in each leaf after dark adaptation, and this measurement was replicated five times. The OJIP curves were induced by 3,000 μmol⋅m^-2^⋅s^-1^ pulsing red light. Then, the relative fluorescence intensity between 10 μs and 1 s was measured and points O, J, I, and P on the OJIP curve corresponded to the time of 0, 2, 30, and 1,000 ms. The relative fluorescence intensity at point O was defined as 0, and that at point P was defined as 1. The OJIP curve was standardized based on the formula V_O-P_ = (F_t_ - F_o_)/(F_m_ - F_o_); then, the relative variable fluorescence at point K (0.3 ms), point J (2 ms), *V*_K_ and *V*_J_ could be obtained. The difference between the standardized OJIP curve of *L. perenne* leaves under different Cd concentrations and in the treatment with no Cd was taken and presented as Δ*V*_O-P_. Then, the JIP-test was conducted on the OJIP curves and the chlorophyll fluorescence parameter was obtained, including maximal photochemical efficiency of PSII (*F*_v_/*F*_m_), photosynthetic performance index based on absorption of light energy (*PI*_ABS_), light energy absorbed by per unit of reaction center (*ABS*/*RC*), the energy for electron transport derived from the light energy absorbed per unit reaction center (*ET*_o_/*RC*), energy dissipated by per unit reaction center (*DI*_o_/*RC*), light energy absorbed per unit area (*ABS*/*CS*_m_), the energy for electron transport derived from the light energy absorbed by per unit area (*ET*_o_/*CS*_m_), and energy dissipated per unit area (*DI*_o_/*CS*_m_). The calculation methods for the abovementioned parameters were based on research conducted by Strasser et al. (1995).

#### Data Processing Methods

Excel and SPSS software were used to conduct statistical analyses on the measured data. The data in the figure shows mean ± SD. A one-way analysis of variance (ANOVA) and least significant difference (LSD) test were adopted to compare the differences between treatments.

## Results and Analysis

### The Effects of Inoculating *L. perenne* With *G. mosseae* on Plant Growth Characteristics in Cadmium Contaminated Soil

**Table 1** shows that the effect of AMF inoculation on root/shoot ratio of *L. perenne* was insignificant; however, the effect on belowground biomass (*P* < 0.01) and other growth parameters was significant (*P* < 0.001). Soil Cd significantly affected the growth parameters of *L. perenne* (*P* < 0.001) but showed no significant interaction with AMF inoculation.

**Table 1:** Two-way ANOVAs examining the effects of AMF inoculation (+AMF), soil Cd (Cd), and their interaction (+AMF × Cd) on plant height, root length, total biomass, aboveground biomass, underground biomass, and root/shoot ratio.

	+AMF		Cd		+AMF × Cd	
			
	*F*	*P*	*F*	*P*	*F*	*P*
plant height	19.15	<0.001	11.71	<0.001	0.55	0.65
root length	51.11	<0.001	128.68	<0.001	1.73	0.20
total biomass	62.61	<0.001	13.56	<0.001	1.78	0.19
aboveground biomass	65.91	<0.001	7.82	<0.001	1.05	0.40
underground biomass	62.81	<0.01	22.56	<0.001	2.83	0.07
root/shoot ratio	6.83	0.02	45.04	<0.001	1.59	0.23


**FIGURE 1:**
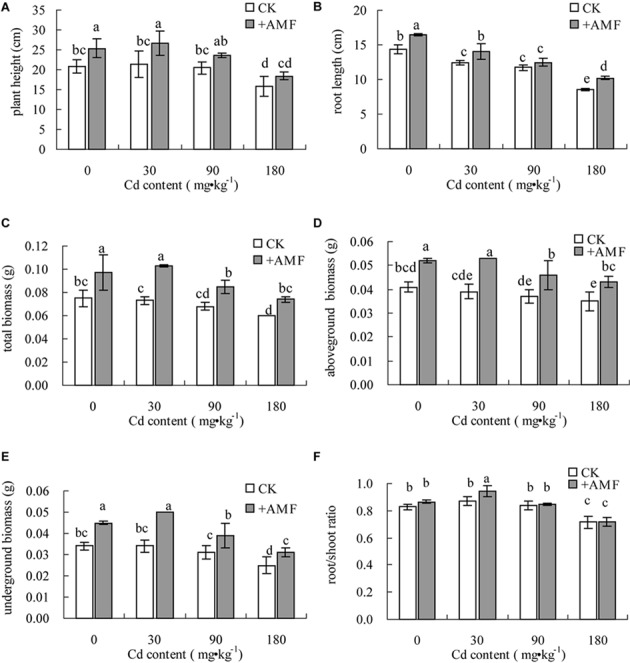
The effects of inoculating *Lolium perenne* with *G. mosseae* on the plant height **(A)**, toot length **(B)**, total biomass **(C)**, aboveground biomass **(D)**, underground biomass **(E)**, and root/shoot ratio **(F)** of the plant in cadmium contaminated soil. Different lowercase letters indicate a significant difference (*P* < 0.05) between the treatment and control.

With the increase of Cd content in the soil, the plant height, root length, and biomass of *L. perenne* presented significantly decreasing trends (**Figures 1A,B**). However, when Cd content was 30 mg⋅kg^-1^, compared to the treatment without Cd stress, *L. perenne* only had a decreased root length, but the plant height and biomass of the different plant parts did not change, and the root to shoot ratio even showed an increasing trend. Under different soil Cd content, the plant height, root length, and biomass of *L. perenne* when inoculated with *G. mosseae* (treatment +AMF) all increased to some extent compared to the CK treatment. When the Cd content in the soil was 180 mg⋅kg^-1^, the plant height, root length, total biomass, and aboveground and underground biomass of *L. perenne* in the treatment +AMF were higher than those in the CK by 16.46% (*P* > 0.05), 18.60% (*P* < 0.05), 23.33% (*P* < 0.05), 22.86% (*P* < 0.05), and 24.00% (*P* < 0.05), respectively (**Figures 1A–E**), but the root shoot ratio of the treatment +AMF had no significant difference compared to the CK (**Figure 1F**).

### Effects of *G. mosseae* Inoculation on the Cd Content in the Underground and Aboveground Parts of *L. perenne* in Cd Contaminated Soil

As shown in **Table 2**, AMF inoculation significantly affected the belowground Cd content of *L. perenne* (*P* < 0.001) but it had no significant effect on aboveground Cd content (*P* > 0.05). Soil Cd showed significant effect on both belowground and aboveground Cd content (*P* < 0.001). The interaction between AMF inoculation and soil Cd had a significant effect on belowground Cd content, but not on aboveground Cd content.

**Table 2:** Two-way ANOVAs examining the effects of AMF inoculation (+AMF), soil Cd (Cd), and their interaction (+AMF × Cd) on Cd content in the underground and aboveground.

	+AMF		Cd		+AMF × Cd	
			
	*F*	*P*	*F*	*P*	*F*	*P*
underground Cd content	85.24	<0.001	581.97	<0.001	10.34	<0.001
aboveground Cd content	0.12	0.90	232.91	<0.001	0.23	0.88


**FIGURE 2:**
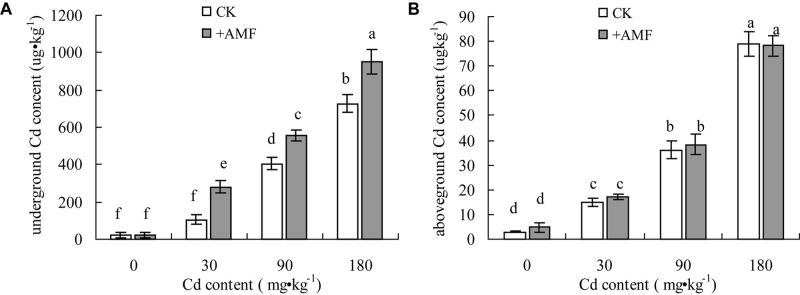
The effects of inoculation with *G. mosseae* on the Cd content in the underground **(A)** and aboveground **(B)** parts of *L. perenne* grown in Cd contaminated soil. Different lowercase letters indicate a significant difference (*P* < 0.05) between the treatment and control.

**Figures 2A,B** show that both the Cd contents in the underground and aboveground parts of *L. perenne* represented significant increasing tendencies with the increasing Cd content in soil, and the Cd content in the underground part of *L. perenne* was higher than in the aboveground plant parts. Inoculation with *G. mosseae* significantly improved the absorption of Cd in *L. perenne* roots. The Cd content in the underground plant parts in the +AMF treatment were significantly higher than the plants not inoculated, except in the treatments without Cd, as there was no significant difference found in the inoculated and non-inoculated treatments. Although *G. mosseae* inoculation improved the absorption of Cd in *L. perenne*, the Cd content in the aboveground part of *L. perenne* in the +AMF treatment was not significantly different from the Cd content in the aboveground part of the non-inoculated *L. perenne*.

### Effects of *L. perenne* Inoculation With *G. mosseae* on Mycorrhizal Infection Rate and Root Vigor in Cadmium Contaminated Soil

The effect of AMF inoculation and soil Cd on mycorrhizal infection rate and root vigor of *L. perenne* was significant (**Table 3**). Moreover, there was a significant interaction effect between these two factors on mycorrhizal infection rate, but not on root vigor (*P* > 0.05).

**Table 3:** Two-way ANOVAs examining the effects of AMF inoculation (+AMF), soil Cd (Cd), and their interaction (AMF × Cd) on mycorrhizal infection rate and root vigor.

	+AMF		Cd		+AMF × Cd	
			
	*F*	*P*	*F*	*P*	*F*	*P*
mycorrhizal infection rate	1046.15	<0.001	16.47	<0.001	13.00	<0.001
root vigor	64.02	<0.001	59.55	<0.001	3.01	0.06


**FIGURE 3:**
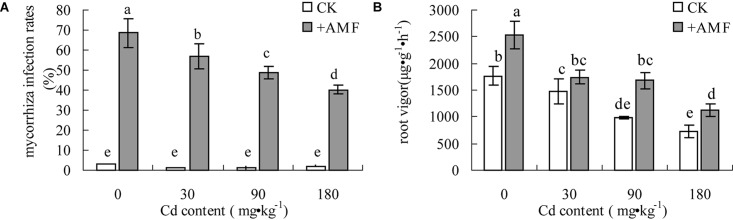
The effects of inoculating *L. perenne* with *G. mosseae* on mycorrhizal infection rate **(A)** and root vigor **(B)** in cadmium contaminated soil. Different lowercase letters indicate a significant difference (*P* < 0.05) between the treatment and control.

The mycorrhizal infection rate of *L. perenne* root systems in the treatment +AMF increased by approximately 50–70%, which presented an obvious decreasing trend with the increase of Cd content in the soil (**Figure 3A**). When the soil Cd content reached 180 mg⋅kg^-1^, the infection rate of the root system in the treatment +AMF declined by 41.31% (*P* < 0.05) compared to the treatment without Cd. The root vigor of *L. perenne* also showed a decreasing trend with the increase of Cd content in the soil (**Figure 3B**). Due to the impact of the *G. mosseae* infection, the root vigor of *L. perenne* in the +AMF treatments under different soil Cd content was higher than that in the treatment without Cd.

### Effects of *L. perenne* Inoculation With *G. mosseae* on the Chlorophyll Content of Leaves From Plants Grown in Cadmium Contaminated Soil

The effects on various chlorophyll contents are shown in **Table 4**. The effect of AMF inoculation on contents of chlorophyll a, total chlorophyll, and chlorophyll a/b of *L. perenne* was significant (*P* < 0.01), but for the chlorophyll b content, it was not significant (*P* > 0.05). Soil Cd had a significant effect on contents of chlorophyll a, chlorophyll b, total chlorophyll, and chlorophyll a/b (*P* < 0.001). However, there was no significant interaction effect between these two factors on chlorophyll contents and ratio of *L. perenne*.

**Table 4:** Two-way ANOVAs examining the effects of AMF inoculation (+AMF), soil Cd (Cd), and their interaction (+AMF × Cd) on chlorophyll a content, chlorophyll b content, total chlorophyll a content, and chlorophyll a/b.

	+AMF		Cd		+AMF × Cd	
			
	*F*	*P*	*F*	*P*	*F*	*P*
chlorophyll a content	102.05	<0.01	143.10	<0.001	0.57	0.64
chlorophyll b content	4.73	0.04	8.65	<0.001	0.12	0.95
total chlorophyll content	51.43	<0.001	76.82	<0.001	0.48	0.70
chlorophyll a/b	11.93	<0.01	12.15	<0.001	0.49	0.69


**FIGURE 4:**
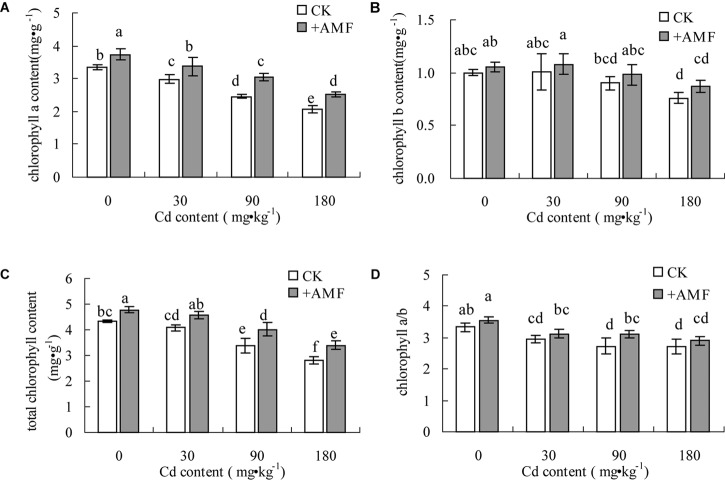
The effects of inoculation with *G. mosseae* on the chlorophyll a content **(A)**, chlorophyll b content **(B)**, total chlorophyll a content **(C)**, and chlorophyll a/b **(D)** of *L. perenne* leaves in cadmium contaminated soil. Different lowercase letters indicate a significant difference (*P* < 0.05) between the treatment and control.

With the increase of soil Cd content, the chlorophyll a, chlorophyll b, and total chlorophyll content of *L. perenne* leaves all declined (**Figures 4A–C**). Moreover, the degree of decrease of the chlorophyll a content was higher than that of chlorophyll b; thus, the chlorophyll a/b value also decreased with the increasing soil Cd content (**Figure 4D**). In the treatments with different soil Cd content, inoculation with *G. mosseae* could increase the chlorophyll content of *L. perenne* leaves; however, the difference in the chlorophyll b content of *L. perenne* leaves between the treatment +AMF and the CK was not significant while both the differences in chlorophyll a and total chlorophyll content reached significant levels. Inoculation with *G. mosseae* could increase the chlorophyll a/b value of *L. perenne* leaves under Cd stress.

### Effects of *G. mosseae* Inoculation on the Photosynthetic Characteristics of *L. perenne* Leaves in Cadmium Contaminated Soil

The effect of AMF inoculation on net photosynthetic rate, stomatal conductance, and transpiration rate of *L. perenne* was significant (*P* < 0.01, **Table 5**). Soil Cd also had a significant effect on these parameters (*P* < 0.001). However, these two factors had no significant effect on intercellular CO_2_ concentration of *L. perenne* (*P* > 0.05) and there was no significant interaction effect between these two factors on the photosynthetic gas exchange parameters.

**Table 5:** Two-way ANOVAs examining the effects of AMF inoculation (+AMF), soil Cd (Cd), and their interaction (AMF × Cd) on net photosynthetic rate, stomatal conductance, transpiration rate, and intercellular CO_2_ concentration.

	+AMF		Cd		+AMF × Cd	
			
	*F*	*P*	*F*	*P*	*F*	*P*
net photosynthetic rate	7.26	<0.01	14.77	<0.001	0.49	0.70
stomatal conductance	11.56	<0.01	27.57	<0.001	0.35	0.79
transpiration rate	10.27	<0.01	8.86	<0.001	1.59	0.25
intercellular CO_2_ concentration	3.91	0.06	4.41	0.02	2.04	0.15


**FIGURE 5:**
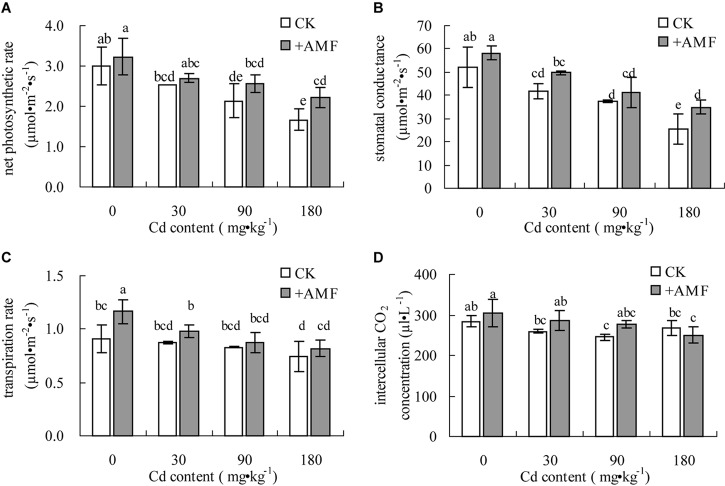
The effects of inoculation with *G. mosseae* on the net photosynthetic rate **(A)**, stomatal conductance **(B)**, transpiration rate **(C)**, and intercellular CO_2_ concentration **(D)** of *L. perenne* leaves in cadmium contaminated soil. Different lowercase letters indicate a significant difference (*P* < 0.05) between the treatment and control.

The photosynthetic gas exchange parameters of *L. perenne* leaves were obviously affected by soil Cd content (**Figure 5**). With the increase of the soil Cd content, the *P*_n_, *G*_s_, and *T*_r_ all presented declining tendencies (**Figures 5A–C**). When the soil Cd content increased to 180 mg⋅kg^-1^, the *C*_i_ of *L. perenne* leaves in the CK increased by 8.98% (*P* > 0.05) compared to the *C*_i_ of the 90 mg⋅kg^-1^ Cd treatment (**Figure 5D**). Inoculation with *G. mosseae* could evidently improve the *G*_s_ of *L. perenne* leaves, increasing both *T*_r_ and *P*_n_. When the soil Cd content increased to 180 mg⋅kg^-1^, the *P*_n_, *G*_s_, and *T*_r_ of *L. perenne* leaves in the treatment +AMF increased by 33.20% (*P* < 0.05), 37.79% (*P* < 0.05), and 10.06% (*P* > 0.05), respectively, compared to CK.

### Effects of *G. mosseae* Inoculation on the OJIP Curves of *L. perenne* Leaves in Cadmium Contaminated Soil

**FIGURE 6:**
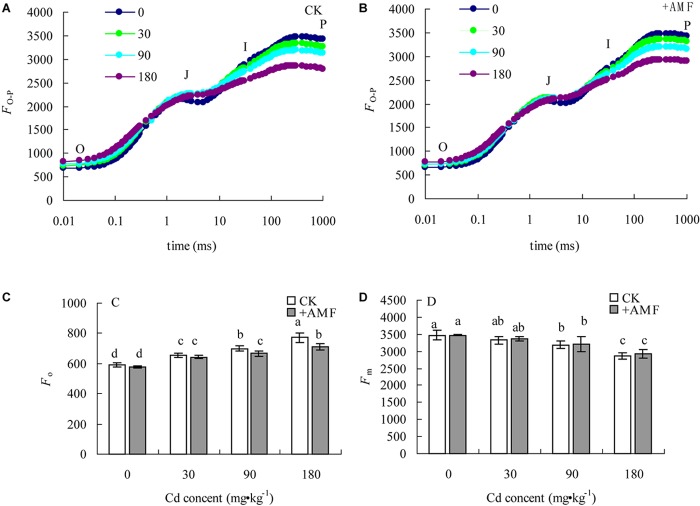
The effects of inoculation with *G. mosseae* on the OJIP curve **(A,B)**, *F*_o_ **(C)**, and *F*_m_ **(D)** of *L. perenne* leaves in cadmium contaminated soil. Different lowercase letters indicate a significant difference (*P* < 0.05) between the treatment and control. **(A,B)** data is the average of five repetitions.

Soil Cd content could evidently influence the shape of OJIP curves in *L. perenne* leaves (**Figures 6A,B**). With the increase of soil Cd content, the relative fluorescence intensity at point O increased, while that at point, P presented an obvious decreasing tendency; the reduction of *F*_m_ was the most significant, and the OJIP curve became smoother with increasing soil Cd content. AMF inoculation had a significant effect on *F*_o_ of *L. perenne* (*P* < 0.001, **Table 6**), but not on *F*_m_ (*P* > 0.05). Soil Cd exerted a significant effect on *F*_o_ and *F*_m_ (*P* < 0.001). However, there was no significant interaction effect between these two factors on *F*_o_ and *F*_m_. In the treatment +AMF, the variation of the OJIP curve in *L. perenne* leaves was obviously smaller in the CK. Quantitative analysis of the relative fluorescence intensity at point O and P (*F*_o_ and *F*_m_) showed that when soil Cd treatments were 0 and 30 mg⋅kg^-1^, the difference of *F*_o_ in *L. perenne* leaves between the treatment +AMF and CK was not significant. However, when the Cd content was 90 and 180 mg⋅kg^-1^, the *F*_o_ of *L. perenne* leaves in the treatment +AMF was significantly lower than in the CK. Although under the Cd content treatments, the *F*_o_ of *L. perenne* leaves in the treatment +AMF were all higher than in the CK, but the difference was not significant (**Figures 6C,D**).

**Table 6:** Two-way ANOVAs examining the effects of AMF inoculation (+AMF), soil Cd (Cd), and their interaction (AMF × Cd) on *F*_o_ and *F*_m_.

	+AMF		Cd		+AMF × Cd	
			
	*F*	*P*	*F*	*P*	*F*	*P*
*F*_ o_	16.70	<0.001	84.42	<0.001	2.59	0.09
*F*_ m_	0.42	0.53	24.60	<0.001	0.10	0.96


### Effects of *G. mosseae* Inoculation on Electron Transport at the Donor and Receptor Sides of PSII of *L. perenne* Leaves in Cadmium Contaminated Soil

**FIGURE 7:**
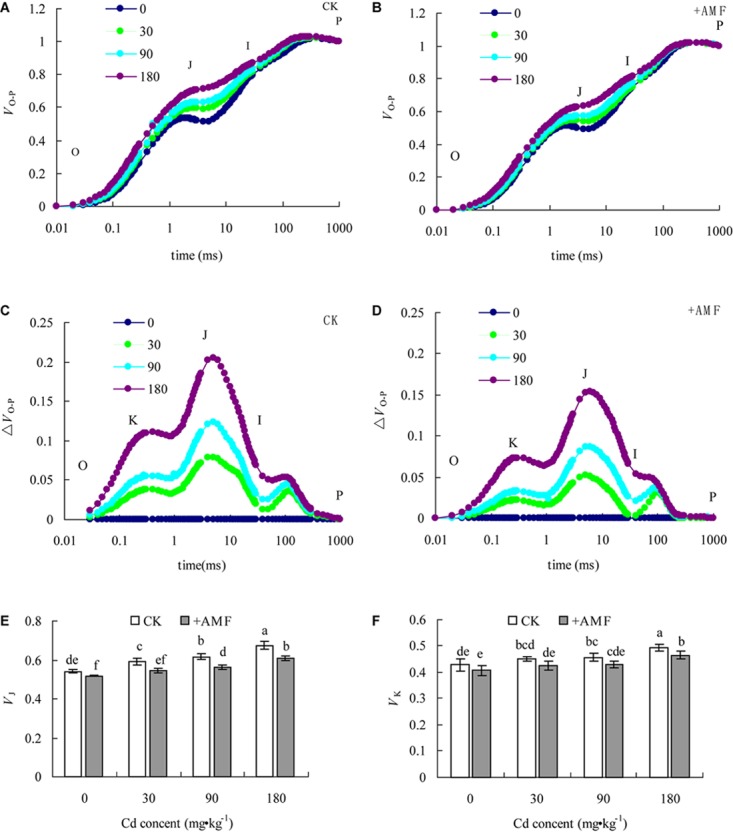
The effects of inoculation with *G. mosseae* on the *V*_O-P_ **(A,B)**, Δ*V*_O-P_ **(C,D)**, *V*_J_ **(E)**, and *V*_K_ **(F)** of *L. perenne* leaves in cadmium contaminated soil. Different lowercase letters indicate a significant difference (*P* < 0.05) between the treatment and control. **(A,D)** data is the average of five repetitions.

The relative fluorescence intensity at point O was defined as 0 and that at point P was defined as 1. After the OJIP curves of *L. perenne* leaves under different treatments were standardized (**Figures 7A–D**), the data showed that with the increase of soil Cd content, *V*_J_ increased, and the relative variable fluorescence at point J (2 ms) had the most obvious increase. However, the increase of *V*_J_ in the treatment +AMF was significantly lower than that in the CK. The standardized OJIP curves under different soil Cd content treatments compared with that in the treatment where the Cd content was 0 showed that in addition to the significantly increased relative variable fluorescence at point J, there was also an evident K point (at 0.3 ms). According to the quantitative analysis of *V*_J_ and *V*_K_ variations, with an increase of soil Cd content, *V*_J_ and *V*_K_ significantly increased and the increase of *V*_J_ was significantly greater than that of *V*_K_.

**Table 7** shows that the effect of AMF inoculation and soil Cd on *V*_J_ and *V*_K_ of *L. perenne* was significant (*P* < 0.001), but their interaction effect on *V*_J_ and *V*_K_ was not significant. In different soil Cd content treatments, *V*_J_ and *V*_K_ of in the treatment +AMF were obviously lower than those in the CK. In addition, in different soil Cd content treatments, the *V*_J_ in the treatment +AMF was significantly lower than in the CK, while the difference in *V*_K_ between the two treatments only reached a significant level when Cd content was 180 mg⋅kg^-1^ in Cd contaminated soil (**Figures 7E,F**).

**Table 7:** Two-way ANOVAs examining the effects of AMF inoculation (+AMF), soil Cd (Cd), and their interaction (AMF × Cd) on *V*_J_ and *V*_K_.

*V*_J_	+AMF		Cd		+AMF × Cd	
			
*V*_K_	*F*	*P*	*F*	*P*	*F*	*P*
*V*_J_	86.25	<0.001	83.96	<0.001	3.44	0.04
*V*_K_	14.81	<0.001	14.95	<0.001	0.05	0.98


### Effects of *G. mosseae* Inoculation on Photochemical Activities of PSII of *L. perenne* Leaves in Cadmium Contaminated Soil

The AMF inoculation and soil Cd had significant effect on *F*_v_/*F*_m_ and *PI*_ABS_ of *L. perenne* (*P* < 0.001), which is shown in **Table 8**, but they had no significant interaction effect on these parameters.

**Table 8:** Two-way ANOVAs examining the effects of AMF inoculation (+AMF), soil Cd (Cd), and their interaction (AMF × Cd) on *F*_v_/*F*_m_ and *PI*_ABS_.

	+AMF		Cd		+AMF × Cd	
			
	*F*	*P*	*F*	*P*	*F*	*P*
*F*_v_/*F*_m_	15.93	<0.001	154.46	<0.001	3.42	0.04
*PI*_ABS_	36.62	<0.001	104.49	<0.001	0.06	0.98


**FIGURE 8:**
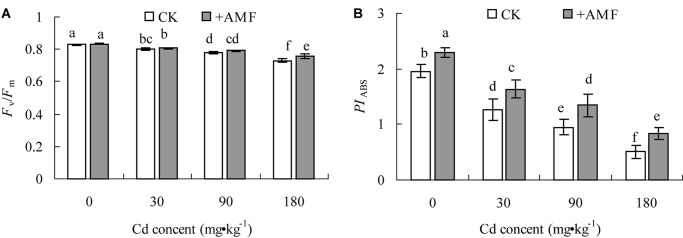
The effects of inoculation with *G. mosseae* on the *F*_v_/*F*_m_ **(A)** and *PI*_ABS_ **(B)** of *L. perenne* leaves in cadmium contaminated soil. Different lowercase letters indicate a significant difference (*P* < 0.05) between the treatment and control.

In soil without Cd contamination, there was no significant difference between *F*_v_/*F*_m_ in the *L. perenne* leaves between the treatment +AMF, and the CK (**Figure 8A**); however, the *PI*_ABS_ of the treatment +AMF exceeded that of the CK by 17.08% (*P* < 0.05), which was significant (**Figure 8B**). With the increase of soil Cd content, the *F*_v_/*F*_m_ and *PI*_ABS_ of the *L. perenne* leaves both presented obvious declining trends; the decrease of *PI*_ABS_ was the greatest. When the soil Cd content was between 0 and 90 mg⋅kg^-1^, the *F*_v_/*F*_m_ in the leaves from *L. perenne* in the +AMF treatment were not significantly different from that in the CK treatment. When the soil Cd content was 180 mg⋅kg^-1^, the *F*_v_/*F*_m_ in the leaves from *L. perenne* in the +AMF treatment increased by 4.82% (*P* < 0.05) compared to the CK, while *PI*_ABS_ in the +AMF treatment under different soil Cd contents were all significant higher than the CK.

### Effects of *G. mosseae* Inoculation on Light Energy Absorption and Utilization Parameters of *L. perenne* Leaves in Cadmium Contaminated Soil

The AMF inoculation showed significant effect on *ABS*/*RC, ET*_o_/*RC, DI*_o_/*RC, ET*_o_/*CS*_m_, and *DI*_o_/*CS*_m_ (*P* < 0.001, **Table 9**), but an insignificant effect on *ABS*/*CS*_m_ (*P* > 0.05). Soil Cd also had a significant effect on these parameters (*P* < 0.001). However, the interaction effect between AMF inoculation and soil Cd on these parameters was not significant.

**Table 9:** Two-way ANOVAs examining the effects of AMF inoculation (+AMF), soil Cd (Cd), and their interaction (AMF × Cd) on *ABS*/*RC, ET*_o_/*RC, DI*_o_/*RC, ABS*/*CS*_m_, *ET*_o_/*CS*_m_, and *DI*_o_/*CS*_m_.

	+AMF		Cd		+AMF × Cd	
			
	*F*	*P*	*F*	*P*	*F*	*P*
*ABS*/*RC*	24.92	<0.001	42.55	<0.001	0.75	0.53
*ET*_o_/*RC*	100.65	<0.001	82.70	<0.001	6.21	0.05
*DI*_o_/*RC*	26.71	<0.001	116.11	<0.001	3.56	0.03
*ABS*/*CS*_m_	0.94	0.34	38.67	<0.001	0.27	0.85
*ET*_o_/*CS*_m_	42.63	<0.001	112.04	<0.001	1.44	0.25
*DI*_o_/*CS*_m_	29.97	<0.001	159.28	<0.001	3.78	0.02


**FIGURE 9:**
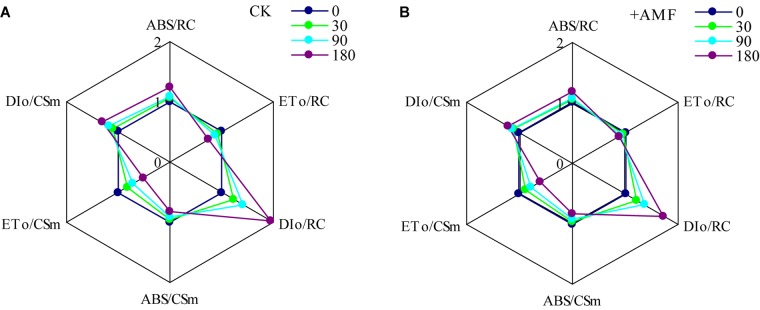
The effects of inoculating *L. perenne* with *G. mosseae* on light energy absorption and utilization parameters **(A,B)** in plants grown in cadmium contaminated soil. **(A,B)** data is the average of five repetitions.

Cadmium (Cd) stress significantly changed the light energy absorption and utilization parameters per unit reaction center and per unit area of *L. perenne* leaves (**Figures 9A,B**). With the increase of Cd content, the *ABS*/*RC* increased evidently, while the *ABS*/*CS*_m_ presented an obvious decreasing trend. Cd stress also resulted in a significant decrease of *ET*_o_/*RC* and *ET*_o_/*CS*_m_ as well as the increase of *DI*_o_/*RC* and *DI*_o_/*CS*_m_; the decrease of *ET*_o_/*CS*_m_ and increase of *DI*_o_/*RC* were much greater. However, inoculating *L. perenne* with *G. mosseae* obviously mitigated the variation of each of the energy allocation parameters in the Cd treatments.

## Discussion

Cadmium (Cd) can result in the suppression of plant growth (Liang et al., 2005). Plants must adapt to adverse environmental conditions both morphologically and physically to maintain normal growth and ensure the normal accumulation of dry mass, thus maintaining normal physical processes such as water absorption by roots and photosynthesis of the aboveground parts (Zhang H.H. et al., 2012). In this experiment, the growth of *L. perenne* in Cd contaminated soil was obviously inhibited; plant height, root length, and biomass accumulation obviously declined with the increase of soil Cd content. However, when soil Cd content was 30 mg⋅kg^-1^, the root to shoot ratio increased to some extent compared to the treatment without Cd, which might be an adaptation to Cd stress. However, with the increase of soil Cd content, the root to shoot ratio gradually decreased, indicating that Cd in the soil would first result in the blockage of *L. perenne* root growth and development. It is possible that *L. perenne* roots would accumulate Cd and alleviate the effects of Cd on aboveground plant growth. The infection level of *L. perenne* roots by *G. mosseae* was relatively high, and due to this infection, the root vigor of *L. perenne* in the treatment +AMF was significantly higher than that in the CK under different Cd contamination levels, hence increasing the root length and root biomass of *L. perenne* grown under Cd stress. Inoculation with *G. mosseae* increased the Cd content in *L. perenne* roots but had no significant effect on the Cd content in aboveground plant parts. This might be related to the Cd immobilization by the AMF or the increased biomass in the aboveground parts of *L. perenne*, which diluted the Cd concentration.

In addition to the toxicity to plant roots, the effects of Cd on plant growth also included the inhibition of plant photosynthesis under relatively high Cd concentrations, which was one of the important reasons for the suppression of plant growth. Excessive Cd absorption in *Phaseolusvul garis* destroyed the structure of the chloroplasts, influenced chlorophyll synthesis or accelerated its degradation rate (Siedlecka and Krupa, 1996), and destroyed mesophyll cells in leaves of rye (*Secale cereale* L. cv. Pastar), increasing the resistance of CO_2_ transport in mesophyll cells, which resulted in the decrease or loss of key enzyme activity for photosynthesis (Krupa et al., 1999). Simultaneously, Cd stress also resulted in the decrease of Rubsico affinity to CO_2_ and the stability of the photosynthetic apparatus as well as the blocking of electron transport along the electron transport chain, inducing free radical accumulation and leading to oxidative damage of cells (Shah et al., 2001; Olmos et al., 2003). In this study, with the increase of soil Cd content, the chlorophyll content of *L. perenne* leaves declined and the chlorophyll a/b value also presented a decreasing tendency, indicating that in *L. perenne* leaves, chlorophyll a was more sensitive to Cd than chlorophyll b. Chlorophyll a served as a light energy receptor and the reaction center for photoreaction in plants, which was responsible for converting light energy to electrical energy and conducting electron transport on the electron transport chain. Therefore, Cd stress resulted in the decrease of the light energy capturing capacity of *L. perenne* leaves and led to a declining light energy utilization capacity. Although the +AMF treatment increased chlorophyll a and b content compared to the CK treatment when grown in soil contaminated with Cd, the chlorophyll b content between the treatments was not significantly different, but the chlorophyll a content was significantly different. This indicates that inoculation with *G. mosseae* improved the light energy acquisition in leaves of *L. perenne* when grown on Cd contaminated soil by increasing the chlorophyll a.

Studies have also shown that Cd can result in disordered stacking of grana, the disappearance of stroma lamella and the decrease of chloroplast function leading to the suppression of the capacity for photosynthetic carbon assimilation in plants (Zhang et al., 2005; Deng et al., 2014). The Cd content in the aboveground parts of the +AMF treatment grown on soil with different Cd contents was not significantly different compared to the CK, but the photosynthetic gas exchange parameters between the two treatments were significantly different. In this study, with the increase of soil Cd content, *P*_n_, *G*_s_, and *T*_r_ presented obvious declining tendencies. Inoculating *L. perenne* with *G. mosseae* evidently improved the stomatal limitation of leaves, hence increasing its *T*_r_ and *P*_n_, which was favorable for the accumulation of assimilates. In the +AMF treatment, the stomatal aperture in *L. perenne* leaves might be related to the change of endogenous hormones, including plant cytokinins, induced by inoculation with AMF (Cosme and Wurst, 2013). When soil Cd content increased to 180 mg⋅kg^-1^, the *C*_i_ of *L. perenne* leaves in the treatment +AMF showed a continuous decreasing trend, while that of the CK increased to some extent compared to plants grown in 90 mg⋅kg^-1^ of Cd. This indicated that soil Cd content of 180 mg⋅kg^-1^ had an inhibiting effect on the photosynthesis of *L. perenne*, which was also related to the destruction of the photosynthetic apparatus in leaves by Cd as well as reducing its CO_2_ utilization ability, and *C*_i_ increased even when stomatal conductance decreased. However, *C*_i_ in the treatment +AMF was lower than that in the CK when Cd content reached 180 mg⋅kg^-1^, indicating that inoculation with *G. mosseae* could protect the physical functions of the photosynthetic apparatus in the host and increase CO_2_ assimilation capacity. Inoculation with *G. mosseae* could improve the photosynthetic capacity of *L. perenne* leaves by increasing the stomatal aperture when plants are grown in soil with a low level of Cd contamination. However, when the soil Cd content was 180 mg⋅kg^-1^ and the non-stomatal factors impacted photosynthesis, inoculation with *G. mosseae* could also improve the photosynthetic capacity through non-stomatal factors including increasing the CO_2_ fixation capability of *L. perenne* leaves. This might be related to the effects of AMF on the activities of some enzymes such RuBP carboxylase/oxygenase (RuBisCO), D-fructose-1,6-bisphosphatase (FBPase), D-fructose-6-phosphatase (F6P), and ribulose-5-phosphatekinase (Ru5PK) (Chen et al., 2017c). Previous studies have found that heavy metal ions could react with nutrients, such as phosphate radicals (HPO_4_^2-^, H_2_PO_4_^-^), resulting in restrictions in absorption of mineral nutrition elements such as phosphorus (Liu et al., 2017). However, inoculation with AMF could improve the plant’s ability to absorb minerals, especially phosphorus (Li et al., 1991; Ahmed et al., 2006; Dong et al., 2008; Xu et al., 2008), which has been shown to play an important role in normal photosynthetic carbon assimilation processes. Therefore, inoculation with *G. mosseae* could improve the photosynthetic carbon assimilation capability of *L. perenne* leaves possibly due to the improvement of the absorption of phosphorus by roots; however, this requires further research.

The chlorophyll fluorescence technique is an important method to study the plant photosynthetic apparatus, especially the function of PSII (Misra et al., 2012; Kalaji et al., 2016, 2017). Heavy metal stress could significantly decrease the PSII activity in leaves and inhibit the electron transfer process (Mathur et al., 2016). In this study, with the increase of soil Cd content, the *F*_o_ on the OJIP curve of *L. perenne* leaves increased, while the *F*_m_ presented a decreasing trend; both *F*_v_/*F*_m_ and *PI*_ABS_, the parameters reflecting PSII photochemical activity of *L. perenne* leaves, evidently declined. *PI*_ABS_ showed a stronger decrease than *F*_v_/*F*_m_, reflecting the sensitivity of this PSII parameter (Kalaji et al., 2014). However, the decrease of *F*_v_/*F*_m_ and *PI*_ABS_ in the +AMF treatment was evidently lower than those in the CK, which indicated that inoculation with *G. mosseae* could mitigate the decrease of PSII photochemical activity in *L. perenne* leaves. To further analyze the reason why inoculation with *G. mosseae* could promote PSII photochemical activity in *L. perenne* leaves under Cd stress, the OJIP curves of the different treatments were standardized. The results showed that, compared to the treatment with a soil Cd content of 0, both the relative variable fluorescence *V*_J_ and *V*_K_ at point J (2 ms) and point K (0.3 ms) increased evidently; *V*_J_ increased in particular. Some studies found that in adverse environmental conditions, the blocking of photosynthetic electron transport usually occurred at the electron receptor side and the electron donor side of the PSII reaction center. At the electron receptor side, transport from *Q*_A_ (primary electron receptor of the PSII electron transport chain) to *Q*_B_ (secondary electron receptor of the PSII electron transport chain) was the main inhibited site, while at the electron donor side, the activity of the oxygen-evolving complex (OEC) was one of the main parts sensitive to adverse environmental conditions (Abbasi and Komatsu, 2004; Park et al., 2004; Zhang et al., 2018). The relative variable fluorescence *V*_J_ at point J (2 ms) on the OJIP curve could reflect the amount of accumulation of *Q*_A_^-^. Specifically, the enhancement of *V*_J_ indicated the blockage of electron transport from *Q*_A_ to *Q*_B_ on the electron receptor side of PSII (Haldimann and Strasser, 1999; Zhang et al., 2016) and the increase of *V*_K_ was considered the specific tag of damaged OEC activity on the electron donor side of PSII (Zhang Z. et al., 2012). Therefore, the decline of PSII photochemical activity in *L. perenne* leaves under Cd stress was mainly related to the inhibition of electron transport from *Q*_A_ to *Q*_B_ on the PSII electron receptor side caused by Cd stress and the reduction of OEC activity in the PSII electron donor side. The PSII receptor side was especially sensitive to Cd stress. However, under different soil Cd treatments, the increase of the *V*_J_ and *V*_K_ in *L. perenne* leaves in the treatment +AMF were significantly lower than in the CK, which showed that inoculation with *G. mosseae* could promote PSII activity through stabilizing electron transport at the PSII receptor and donor sides. Inoculation with *G. mosseae* could improve the PSII function in *L. perenne* leaves by improving the absorption of nutrients, reducing the distribution of heavy metals in the aboveground part of the host plants (Lins et al., 2006; Chen and Zhao, 2009; Słomka et al., 2011; Zhang H.H. et al., 2012), which might also be related to the effects of AMF on the activities of some enzymes and hormones in the host plants or the induction on the expressions of related resistance genes which further initiate the anti-oxidation system. For example, inoculation with *G. mosseae* could significantly improve the activities of secondary metabolism-related enzymes including polyphenol oxidase, peroxidase, and phenylalanine ammonia-lyase in *Cabernet sauvignon* roots, alleviating injuries to plant cell membranes caused by Cd stress (Qu et al., 2009). Infection with AMF could induce a significant increase in the content of osmotic adjustment substrates, such as proline, in *Coffea arabica* leaves (Andrade et al., 2010). Inoculation with *Glomus intraradices* and *G. mosseae* could induce the expression of heavy metal stress-related genes in *Populus alba* plants grown in soil contaminated with heavy metals and increase the polyamine level (Cicatelli et al., 2010). In addition, inoculation with *G. mosseae* increased the biomass of *L. perenne*plants (**Figure 1**) and decreased the Cd concentration in *L. perenne* leaves, which might also play an important role in reducing the photoinhibition caused by Cd stress. However, the absorption and distribution mechanism of Cd in *L. perenne* plants through inoculation with *G. mosseae* requires further studies.

Under stress, plants generally improve their adaptability through adjusting energy distribution (Misra et al., 2001). Soil Cd stress evidently changed light energy absorption and utilization parameters per unit reaction center and per unit area of *L. perenne* leaves. With the increase of soil Cd content, although the light energy absorbed per unit area, *ABS*/*CS*_m_, presented an obvious declining trend, while the light energy absorbed per unit of the reaction center, *ABS*/*RC*, increased. This indicates that with the increase of the Cd content, the amount of active reaction centers per unit area of *L. perenne* leaves declined to some extent, forcing the enhancement of the remaining active reaction centers. Cd stress might result in the inactivation of partial reaction centers in *L. perenne* leaves, which was also a type of adaptation mechanism for PSII reaction centers adapting to soil Cd stress (Mlinariæ et al., 2017; Zhang et al., 2017). Nevertheless, Cd stress resulted in the significant decrease on the proportion of light energy absorbed by *L. perenne* leaves used for photochemical reactions, while the proportion of waste heat dissipation increased greatly. However, inoculation with *G. mossea*e significantly mitigated each energy allocation parameter of *L. perenne* leaves under Cd stress. This showed that inoculation with *G. mosseae* could promote PSII photochemical activity and helped the host to adapt to Cd stress through optimizing light energy allocation and utilization in *L. perenne*.

## Conclusion

Soil Cd pollution will first influence *L. perenne* roots, resulting in the decline of root vigor and the decrease of underground biomass accumulation as well as the decline of chlorophyll content and photosynthetic capacity of leaves. Inoculation with *G. mosseae* slightly increased the Cd content in *L. perenne* roots but did not aid in the transport and distribution of Cd to the aboveground parts of *L. perenne*. Inoculation with *G. mosseae* can significantly increase the root vigor and chlorophyll content of *L. perenne* in Cd contaminated soil. In addition, the photosynthetic carbon assimilation capacity of *L. perenne* leaves and PSII photochemical activity also increased to different degrees. Therefore, inoculating *L. perenne* with *G. mosseae* can promote the host’s tolerance to Cd via morphological characteristics and photosynthetic functions as well as reduce the toxicity of Cd to *L. perenne*.

## Author Contributions

NX, HaZ, and HuZ conceived and designed the experiments. All the authors performed the experiments and analyzed the data. NX and HuZ wrote the manuscript and prepared the figures and/or tables. NX, HuZ, HaZ, and SG reviewed drafts of the paper.

## Conflict of Interest Statement

The authors declare that the research was conducted in the absence of any commercial or financial relationships that could be construed as a potential conflict of interest.
